# Epigallocatechin-3-gallate ameliorates radiation-induced acute skin damage in breast cancer patients undergoing adjuvant radiotherapy

**DOI:** 10.18632/oncotarget.9495

**Published:** 2016-05-20

**Authors:** Wanqi Zhu, Li Jia, Guanxuan Chen, Hanxi Zhao, Xiaorong Sun, Xiangjiao Meng, Xianguang Zhao, Ligang Xing, Jinming Yu, Meizhu Zheng

**Affiliations:** ^1^ Department of Radiation Oncology, Shandong Cancer Hospital affiliated to Shandong University, Shandong Academy of Medical Science, Jinan, Shandong, China; ^2^ Shandong Key Laboratory of Radiation Oncology, Jinan, Shandong, China; ^3^ Department of Radiation Oncology, Jinan Fourth People's Hospital, Jinan, Shandong, China; ^4^ Department of Radiology, Shandong Cancer Hospital affiliated to Shandong University, Shandong Academy of Medical Science, Jinan, Shandong, China

**Keywords:** epigallocatechin-3-gallate, breast neoplasms, dermatitis, radiation

## Abstract

There are few effective treatment options for radiation-induced dermatitis in breast cancer patients. We conducted a single-arm trial to tested the hypothesis that topical epigallocatechin-3-gallate (EGCG) is effective against radiation-induced dermatitis in breast cancer patients undergoing radiotherapy. Forty-nine patients participated in this study. The patients underwent mastectomy followed by adjuvant radiotherapy. Topical EGCG was applied daily, starting when grade I dermatitis appeared and ending two weeks after radiotherapy. The maximum dermatitis observed during the EGCG treatment was as follows: Grade 1 toxicity, 71.4% (35 patients); grade 2 toxicity, 28.6% (14 patients); there were no patients with grade 3 or 4 toxicity. The majority of the radiation-induced dermatitis was observed 1 week after the end of radiotherapy. EGCG reduced the pain in 85.7% of patients, burning-feeling in 89.8%, itching in 87.8%, pulling in 71.4%, and tenderness in 79.6%. These findings suggest topical EGCG may be an effective treatment for radiation-induced dermatitis and has acceptable toxicity.

## INTRODUCTION

Despite technological advances, acute radiation skin toxicity (ARST) is the most common side effect of breast cancer radiotherapy, occurring in more than 90% of patients [1]. Complications such as pain, discomfort, irritation, itching, and burning-feeling may cause restriction in movement, unplanned treatment interruptions, and a decreased chance of getting an effective dose. These issues might reduce patients survival rates, as well as their quality of life (QOL) [2, 3].

Epigallocatechin-3-gallate (EGCG) facilitates the healing process in ultraviolet radiation-induced erythema in human skin [4]. Recent study has demonstrated that EGCG enhances viability of human skin cells and decreases apoptosis induced by X-ray irradiation [5]. In addition, recent studies from our laboratory have indicated that oral administration of EGCG protects esophageal epithelial cells from damage [6, 7].

Our recent phase I study has demonstrated that the topical administration of EGCG is safe, and that the recommended concentration is 660 μmol/L during skin radiation [8]. In this prospective study, we carried out a single-institution phase II trial to assess the effectiveness of EGCG as a topical agent for ARST, and to evaluate the radiation-induced dermatitis outcomes in women who underwent adjuvant radiotherapy (RT) for breast cancer.

## MATERIALS AND METHODS

### Patients

Patients with modified radical mastectomy receiving external beam RT to chest wall were included in this study. Eligible patients were required to meet the following criteria: age≥18 years; ECOG PS 0-1; no prior radiation to the thorax; adequate hematologic values (granulocytes ≥2,000/ml, platelets ≥100,000/ml, hemoglobin >8 gm/dl), hepatic function (bilirubin <1.5 normal), and renal values (creatinine clearance >50 ml/min). The exclusion criteria were as follows: positive deep margins; pregnancy or lactation; a known allergy or hypersensitivity to EGCG. The patients with large planning target volumes (PTV), sharp surface irregularities (a sudden change in the PTV depth), or an irregular contour (i.e. axillary folds, or inframammary folds), were also excluded. This study was approved by the Institutional Review and Ethical Committees and registered at ClinicalTrials.gov (NCT01481818). Informed consent was obtained from all patients.

### Radiotherapy

Radiation treatment was delivered to the chest wall, including the surgical scar and regional lymph nodes, i.e., supraclavicular and infraclavicular nodes. Chest wall field arrangement included the area between the midsternal line medially, midaxillary line laterally, 2 cm below the contralateral inframammary fold inferiorly, and supraclavicular–axillary field superiorly. The field arrangement involved an anterior photon field against the supraclavicular and infraclavicular regions and an anterior electron field against the chest wall. All patients underwent simulation for verification of the irradiated fields and determination of chest-wall thicknesses. Additional boluses were used in the light of chest-wall thickness variation. The electron energy depended on the chest-wall thickness in the midplane (range, 6–12 MeV). RT was fractioned in 2 Gy five days a week up to 50 Gy [9].

### EGCG administration

The treatment with EGCG solution was given to all patients undergoing RT immediately after grade I toxicity was documented (according to RTOG). The EGCG dose of 660 μmol/L (purity≥95% by HPLC; from NINGBO HEP Biotech Co., Ltd) was chosen based on our previous study [8]. The solution was sprayed by the same investigator three times a day at 0.05 ml/cm^2^ to the whole radiation field for two weeks after radiation completion. The EGCG dose was calculated independently by two investigators; the average of the two measurements was used as the actual dose. EGCG solution was poured into sterilized throat medical sprayer, and uniformly sprayed on the skin from a distance of 10-20 cm from the skin. The skin folds, such as armpits required full stretch and exposure before spraying. Patients followed general good skin-care practices at the start of radiation therapy. They were also advised not to use deodorants, lotions, creams, perfumes, or any other products on the area during the course of radiation therapy.

### Toxicity evaluation

EGCG was administered immediately when Grade 1 dermatitis was diagnosed. The dermatitis progression was recorded weekly until 2 weeks after the end of RT. Evaluation consisted of 1) provider-assessed toxicity assessment using a grading scale following the Radiation Therapy Oncology Group (RTOG) scoring system and 2) patients-reported symptoms measured using the Skin Toxicity Assessment Tool (STAT). The STAT is regarded as a valid and reliable skin-specific assessment tool [10]. The STAT scores range from 0, representing no symptoms, to 5, representing the worst symptoms (pain, burning-feeling, itching, pulling and tenderness). All staff involved in the study received education on how to use the scale. The dermatitis was scored by two radiation oncologists with at least 8 years of experience. If the two scores were inconsistent, another senior doctor would evaluate the dermatitis. Toxicity was evaluated before and three hours after EGCG application, because the half-life of EGCG is about 3 hours [11]. Toxicity of EGCG was graded using the NCI Common Terminology Criteria for Adverse Events (CTCAE) version 3.0 [12].

### Statistical methods

We calculated the estimated sample size using the per-protocol patient population based on the study by Fenig et al, reporting about 12-17% Grade 3-4 dermatitis probability in patients with 50 Gy skin radiation dose [13]. We reasoned that a probability of 17% would be unacceptably high, while a probability of 4% would be very promising from the clinical point of view. Assuming a significance level of 0.05 (2-sided), 49 assessable patients were needed to distinguish a Grade 3-4 radiation dermatitis rate of 4% from an alternative rate of 17% with 80% power. The differences in the scores before, during and after treatment were tested using paired t-tests. The chi-square test was used to examine differences with categorical variables. SPSS (version 17.0; SPSS Inc., Chicago, IL) was used for statistical analyses. All statistical tests were two-sided. Values or P < 0.05 were considered significant.

## RESULTS

The study started in August 2013, and ended in September 2014. Forty-nine eligible patients were included in the study. Characteristics of the forty-nine patients are listed in Table 1.

**Table 1:** Patient demographics and disease characteristics

	No. of Patients (N=49)	%
Age(years)		
Median	45	
Range	22-64	
Smoking status		
Yes	14	28.6
No	35	71.4
Performance status (ECOG)		
0	23	46.9
1	26	53.1
T stage		
T1	12	24.5
T2	27	55.1
T3	9	18.4
T4	1	2.0
N stage		
N0	1	2.0
N1	5	10.2
N2	38	77.6
N3	5	10.2
AJCC stage		
IIIA	44	89.8
IIIB	1	2.0
IIIC	4	8.2
Surgery to		
Right breast	23	46.9
Left breast	26	53.1
Histology		
Invasive ductal carcinoma	44	89.8
Invasive lobular carcinoma	5	10.2

**Figure 1:**
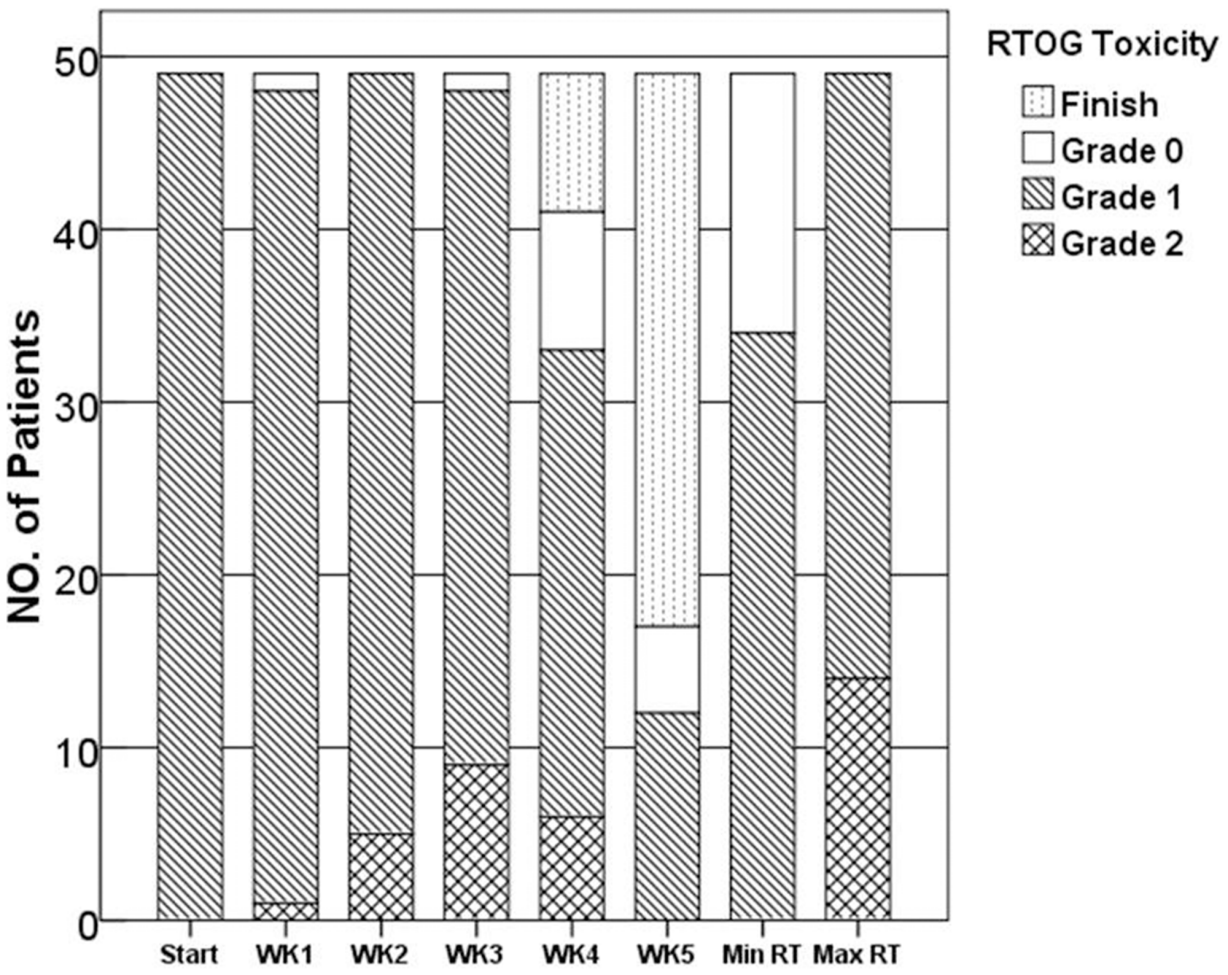
Skin reaction sores during EGCG treatment, as determined by the RTOG scoring system Start=at the beginning of the EGCG therapy; WK1-5=after the first to fifth week of EGCG therapy; Max/Min RT=maximum/minus skin reaction scores during the course of EGCG therapy.

RT was delivered without treatment delays or interruptions to all 49 patients. Grade I dermatitis appeared in 17 patients during the second week of RT, in 24 patients during the third week, and in 8 patients during the fourth week. The mean duration of EGCG treatment was 4 weeks. The maximum radiation-induced skin toxicity observed during the course of EGCG treatment was as follows (Figure 1): Grade 1 toxicity, 71.4% (35 patients); grade 2 toxicity, 28.6% (14 patients); there were no patients with grade 3-4 dermatitis during the EGCG treatment. Importantly, 15 patients exhibited the personal minimum skin toxicity Grade 0. There was a significant difference between the onset and the end of the study in RTOG scores (N=49, t=4.38, p<0.05). The RTOG score was not increased during RT in 35 out of 49 patients.

**Figure 2:**
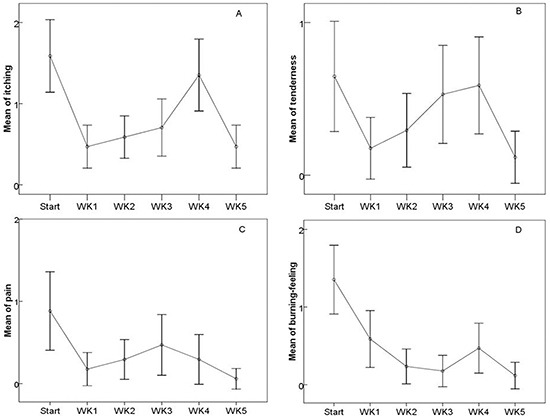
The changes of the patient-reported symptom scores during and after the treatments **A.** Itching, **B.** Tenderness, **C.** Pain, **D.** Burning-feeling.

The patient-reported symptoms assessed by STAT were compared before and after the EGCG treatment (Figure 2). The mean scores of pain, burning-feeling, itching, pulling and tenderness were 0.86±0.82, 1.41±0.70, 1.92±0.93, 0.94±0.72 and 0.92±0.98 at baseline, respectively. Rapid relief of toxicity (patient-reported symptom scores) was observed within 1 week of EGCG treatment in all patients (pain: t=5.229, p<0.05; burning-feeling: t=7.167, p<0.05; itching: t=9.478, p<0.05; pulling: t=2.14, p<0.038, and tenderness: t=4.499, p<0.05). Patient-reported symptom scores were also significantly decreased at the end of the study; pain, burning-feeling, itching, and tenderness scores were t=3.347 p=0.004; t=6.126 p<0.05; t=4.968 p<0.05; t=3.043 p=0.008, respectively. The regression of patient-reported symptoms related to ARST did not seem to depend on the onset time of EGCG. Our data indicate that EGCG has the ability to significantly and persistently control the symptoms of pain (85.7% of patients), burning-feeling (89.8%), itching (87.8%), pulling (71.4%), and tenderness (79.6%). No reported acute toxicity was associated with EGCG.

## DISCUSSION

In this phase II trial, EGCG was applied when patients developed Grade 1 dermatitis [14]. According to a previous study, about 12-17% of patients with 50 Gy skin radiation dose develop Grade 3-4 radiation dermatitis [13]. In our study, no Grade 3-4 dermatitis was found in patients who received the EGCG therapy, and most patients only suffered Grade 1 toxicity, even though they received the same radiation dose. Moreover, EGCG was well tolerated by patients, as we have previously demonstrated in phase I study [8]. The current study provides the clinical evidence for topical EGCG treatment to minimize radiation dermatitis.

Randomized controlled studies of skin care products compared with placebo had generally been negative in preventing or treating radiation dermatitis [15–19]. Recently, Miller et al has suggested that topical monetasone reduces ≥ Grade 3 radiation-induced dermatitis and alleviates the symptoms. However, there was no statistical difference between the mean maximum grades of radiation toxicity [20]. In addition, the side effects of monetasone, including periorificial dermatitis, skin atrophy, and mycotic infection also limited its application in clinic. Therefore, the evidence-based standard of care remained unclear.

A retrospective study demonstrated that topical green tea extracts helped to restore skin integrity in patients with Grade ≥ 2 radiation-induced dermatitis receiving head and neck radiotherapy [21]. Our previous phase I trial also demonstrated that symptoms of radiation-induced dermatitis were alleviated in most patients receiving EGCG (24 patients, 40-660 μmol/L), even during continued RT treatment [8]. Therefore, the efficacy of EGCG for treating radiation dermatitis warranted systematic studies.

EGCG has a role in scavenging superoxide anions, hydroxyl radicals, and hydrogen peroxide [22, 23]. EGCG protects deoxyribonucleic acid against radiation-induced damage through intercalating into DNA, binding to free radicals (FR), and repairing the FR-induced injury. EGCG can inhibit activity of proteasome, which is a key regulator of inflammation, thus inhibiting expression of pro-inflammatory cytokines, such as IL-1β, IL-6, IL-8, and TNFα [24]. Moreover, tea polyphenols have been shown to suppress activity of the proteasome dependent transcription factor NF-B by inhibiting the p38 mitogen-activated protein kinase pathway and IκB kinases [21, 25, 26]. Therefore, the molecular mechanisms underlying the beneficial effects of EGCG in acute radiation-induced skin toxicity are complex and involve antibacterial and anti-inflammatory processes [21].

There are some limitations in the study. First, the dosimetry of the radiation region could not be ensured without three-dimensional planning radiotherapy. Therefore, future phase II randomized controlled trials should use techniques to improve the homogeneity of the planning target volumes. Second, the self-restore capacity of skin might also contribute to the positive result. The prospective randomized, place-controlled design is warranted in future studies.

Based on the clinical data from this trial, topical administration of EGCG solution appears to be an effective treatment for radiation-induced dermatitis in breast cancer patients after mastectomy.
